# Musculoskeletal disorders in physically active conscripts: a one-year follow-up study in the Finnish Defence Forces

**DOI:** 10.1186/1471-2474-10-89

**Published:** 2009-07-22

**Authors:** Henri Taanila, Jaana Suni, Harri Pihlajamäki, Ville M Mattila, Olli Ohrankämmen, Petteri Vuorinen, Jari Parkkari

**Affiliations:** 1 Tampere Research Centre of Sports Medicine, The UKK Institute, Tampere, Finland; 2 Research Department, Centre for Military Medicine, Helsinki, Finland; 3 General Headquarters of Finnish Defence Forces, Helsinki, Finland; 4 Staff Department, Pori Brigade, Säkylä, Finland; 5 Research Unit of Pirkanmaa Hospital District and Department of Trauma, Musculoskeletal Surgery and Rehabilitation, Tampere University Hospital, Tampere, Finland

## Abstract

**Background:**

Musculoskeletal disorders (MSDs) are an important cause for morbidity in military service. They result in disabilities needing long-term rehabilitation and functional impairment leading to premature discharge from military service. The purpose of the study was to investigate the incidence and nature of MSDs in Finnish conscripts.

**Methods:**

Two successive arrivals of 18–28-yr-old male conscripts (*N *= 955, median age 19) were followed for six months. MSDs, including overuse and acute injuries, treated at the garrison clinic were identified and analysed.

**Results:**

During the 12-month study period there were 437 outpatient clinic visits in 955 persons. The occurrence rate was 33% during 6-month service while the event-based incidence was 3.3 per 1000 person-days. Occurrence peaked in summer months. The most common types of MSDs were low back pain (LBP, 20%), lower limb overuse injuries (16%) and sprains or strains (13%). Disorders mostly occurred in combat training in combat gear (40%) and during marching on foot or bicycle (28%). Overuse-related MSDs were more prevalent (66%) than traumatic ones (34%). One-third (34%) of the MSDs were recurrent and 66% were new ones. Disorders of the back and the knee were most frequently recurrent conditions (44% for both). Fractures, knee ligament ruptures, dislocations and muscle strains accounted for the highest number of service days lost. Twenty-four (2.5%) out of 955 conscripts were prematurely discharged due to MSDs.

**Conclusion:**

Preventive measures during military service should be targeted at decreasing low back pain and lower limb overuse injuries, because these inflict the largest burden of MSDs and tend to have a chronic nature.

## Background

Current recommendations for physical activity and public health strongly suggest that engaging in regular physical activity improves cardiovascular health and reduces the risk of many chronic diseases [1]. However, with increasing amounts of physical activity, such as after arrival to military service, there is also an increased risk of musculoskeletal injury or disorder. A recently published hospital discharge register-based study reported an annual incidence for traumatic injury hospitalisation of 94 per 1000 conscripts over a 10-year study period, and concluded that injuries represent a major cause of morbidity in the Finnish Defence Forces. A limitation of the study was, however, that minor injuries not needing hospitalisation were not registered [2]. MSDs represent the second biggest reason for untimely discharge from military service in Finland, and their number rose heavily (62%) at the turn of the millennium [3]. Since over 80% of the male citizens in Finland complete their compulsory military service, musculoskeletal injuries and disorders during military service have also importance from the public health point of view. They result in disabilities needing expensive treatment, long-term rehabilitation and functional impairment leading to premature discharge from military service.

In spite of the overall high prevalence of injuries, there is not much epidemiological data concerning injuries during conscription military service. In addition to hospital discharge studies [2,4], some specific conditions in small target populations have been described such as acoustic injuries [5], frostbites [6], patellar dislocations [7], low back pain (LBP) [8] and stress fractures [9-13]. In the Norwegian and Danish conscription armies, some larger scale epidemiological studies have shown that a significant number of training days are lost due to injuries [14-16]. Before a measure or programme for injury prevention is initiated, the extent of the problem should first be defined. The purpose of this prospective one-year follow-up study was to investigate the incidence and nature of MSDs leading a conscript to seek medical care.

## Methods

### Subjects

The subjects of this study consisted of male conscripts (*N *= 955) from four companies of one brigade (Pori Brigade, Säkylä) in the Finnish Defence Forces. The four companies enrolled into the study were the anti-tank company, the signal company, the mortar company and the engineer company. The Pori Brigade is a typical garrison in the Finnish Defence Forces and the chosen companies form a representative sample of conscripts. During the study year, two arrivals of conscripts started service in the brigade: 359 in July 2006 and 604 in January 2007. Key characteristics of the two arrivals are presented in Table 1.

**Table 1:** Baseline characteristics of two arrivals of 955 male conscripts.

Variable	1st arrival (N = 359)		2nd arrival (N = 596)		Missing (total number)	P-value^1^
Age^2 ^(range yrs)	20 (18–28)		19 (18–27)		3 (0%)	< 0.001
BMI^3 ^(range, kg/m^2^)	23.7 (15.1–45.9)		23.8 (16.4–39.4)		88 (9%)	0.70
	Yes	No	Yes	No		
						
High level of education^4^	151 (42%)	201 (56%)	239 (40%)	351 (59%)	13 (1%)	0.47 ^8^
High level of previous physical activity^5^	114 (32%)	238 (66%)	177 (30%)	412 (69%)	14 (1%)	0.45 ^8^
Good self-assessed health^6^	194 (54%)	158 (44%)	308 (52%)	282 (47%)	13 (1%)	0.39 ^8^
Clear musculoskeletal symptoms^7^	100 (28%)	251 (70%)	174 (29%)	416 (70%)	14 (1%)	0.74 ^8^

1 P-value for difference between the arrivals

2 P-value was examined by using Mann-Whitney U test for median difference

3 Body mass index, P-value was examined by using Independent t test for mean difference

4 Graduated or studies in higher education institution

5 Sweating exercise at least three times per week during the last month before entry to military

6 Compared to age-mates

7 Symptoms lasting more than 7 days at least in one anatomical region during the last month before entry to military

8 P-value was examined by using χ^2 ^statistics for difference

The health status of conscripts was checked during the first week of service by routine medical screenings performed by a physician. If a conscript was found to have had onset of a severe MSD before the beginning of the service, he was discharged. One participant released temporarily (for 24 months) from the service at the medical screening was excluded. Seven (< 1%) out of 962 conscripts refused to participate in the study. All the remaining conscripts agreed to participate and gave their informed consent before the initiation of the study. The age of the conscripts varied from 18 to 28 years (median 19 yr). All subjects were followed for six months starting from the first day of service. Approval for the study protocol was obtained from the Ethical Committee of Pirkanmaa Hospital District on the April 11, 2006 (ref: R06063).

### Physical training programme

In the beginning of military service, all Finnish conscripts perform the basic training of 8 weeks of varying physical activities including marching, cycling, skiing, orienteering, swimming, drill training and combat training in combat gear. There is an average of 17 hours per week of military training and the intensity is constructed so as to be gradually increasing. In addition, conscripts perform other physical exercises such as jogging, team sports, and circuit training 7 hours per week on average. The basic training period is followed by diverse individual training programmes. However, over the following 4 months of service, the amount of moderate and high-intensity physical training is maintained at the same level in different companies. During the first 6 months of military service, conscripts are expected to complete approximately 450 hours of instructed physical training (19 hours per week).

In addition to the compulsory, supervised training garrisons offer a variety of opportunities for physical activity during leisure time including jogging, weight training and lifting and team sports. Approximately 20% to 40% of conscripts practice sports during their leisure time.

### Musculoskeletal disorder registration

The data was collected between July 2006 and June 2007. A musculoskeletal disorder (including overuse and acute injuries) was defined as an event that resulted in physical damage to the body and for which the conscript sought medical care from the garrison clinic. At the clinic, assisted by the healthcare personnel, a conscript filled out a disorder questionnaire eliciting the type, anatomical location, severity, associated activities and cause of MSD. By using this form, minor injuries that would not have been detected by standard medical record data were also identified and analysed. All answers were checked by nurse or physician and any unanswered question was answered if possible. The proportion of unanswered questions was low (< 4% per question). Since conscripts may have had suffered from multiple MSDs during a single visit to the garrison clinic, the total numbers of MSDs exceeded the number of outpatient clinic visits.

The disorder questionnaire included 26 different defined MSD types and an open question for undefined MSD. The type of MSD was categorised as acute if the MSD had sudden onset involving known trauma. Overuse-related MSDs had a gradual onset without known trauma [17,18]. For instance, overuse conditions of the knee, shin, ankle and foot were categorised as lower limb overuse injuries, whereas sprains, strains, wounds, internal knee ligament ruptures and joint dislocations were typically categorised as acute injuries. LBP was defined to be either local pain in the lower back or pain radiating above the knee. The MSD was considered recurrent when the conscript has previously sustained an MSD of the same type and in the same location [17,18].

**Figure 1:**
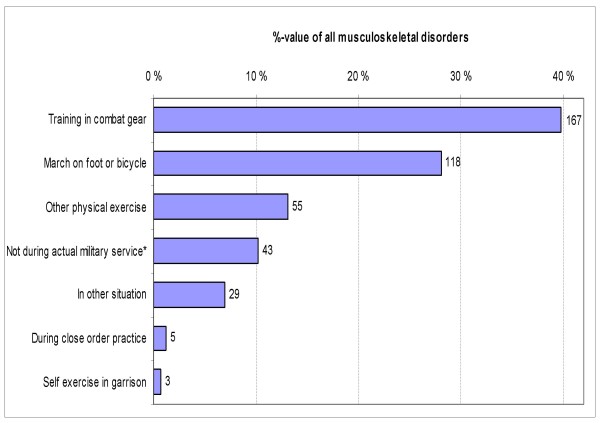
**Distribution of musculoskeletal disorders by associated activities in 955 male conscripts during 6-month military service**. * The term "not during actual military service" includes disorders during vacations, during travel to vacation or back to garrison or during off-duty time in the evenings. Count next to the bar is the absolute number.

Disorders which had occurred during the conscript's leisure time or on the way to vacation or back to garrison were included, but those occurring prior to the beginning of the military service were excluded from the data. The aetiological circumstances of the onset of MSDs during actual military service were charted more thoroughly by use of an additional question (Fig. 1). After careful clinical examination and necessary diagnostic tests and radiological graphs the most accurate diagnosis was selected by a physician according to the 10th Revision of the International Statistical Classification of Diseases and Related Health Problems (ICD-10). The severity of MSD was categorised according to the number of days it prevented physical exercise: 1–7 days denoting minor, 8–30 days moderate and > 30 days severe disorder [19]. Premature release from military service was indicated when a physician determined a conscript unable to continue military training. There were three discharge categories: A) temporary medical discharge from military service; B) permanent medical discharge from service in peacetime; and C) applying for non-military service (Table 2).

**Table 2:** Numbers and reasons for premature discharge from military service.

**A.**	**Reasons for temporary medical discharge from military service**	
	**Number**	**Diagnosis**
	
	**Mental and behavioural disorders**	
	15	Adjustment disorders
	4	Depressive episodes
	3	Anxiety disorders
	1	Mental and behavioural disorders due to use of stimulants
	**Total 23 conscripts, 26% of all premature discharges**	
		
	**Musculoskeletal disorders & injuries**	
	8	Overuse injury of the limb
	3	Tendinopathies
	3	Dislocations
	3	Low back pain
	2	Juvenile osteochondrosis
	2	Internal injury of the knee joint
	1	Fracture of the neck of the femur
	1	Fracture of carpal bones
	1	Injury of the extensor muscle and tendon of a finger
	**Total 24 conscripts, 27% of all premature discharges**	
		
	**Diseases of the respiratory system**	
	**Total 11 conscripts, 12% of all premature discharges**	
		
	**Cardiovascular disorders**	
	**Total 3 conscripts, 3% of all premature discharges**	
		
	**Gastrointestinal diseases**	
	**Total 2 conscripts, 2% of all premature discharges**	
		
	**Dermatological diseases**	
	**Total 2 conscripts, 2% of all premature discharges**	
		
	**Other reasons**	
	1	Sleep disorders
	1	Postviral fatigue syndrome
	1	Pronounced myopia
	**Total 3 conscripts, 3% of all premature discharges**	
	
	**Total 68 conscripts, 76% of all premature discharges**	
		
**B.**	**Reasons for permanent medical discharge from military service**	
	
	**Mental and behavioural disorders**	
	2	Adjustment disorders
	2	Depressive episodes
	1	Mixed and other personality disorders
	1	Panic disorder
	**Total 6 conscripts, 7% of all premature discharges**	
		
**C.**	**16 persons (18% of all premature discharges) applied for non-military service**	

Cases are divided in temporary (A) and permanent (B) categories in 955 male conscripts during a 6-month period, including 16 conscripts who applied for non-military service (C).

### Statistical analysis

SPSS 16.0 for Windows software (SPSS Inc., Chicago, IL) was used for statistical analysis. Occurrence rate was calculated by dividing the number of conscripts with one or more MSDs treated in the garrison clinic (numerator) for MSD by the total number of conscripts (denominator) and expressed as a percent. Person-based incidence was calculated by dividing the number of conscripts treated in the garrison clinic for MSD by the exposure time. Exposure time for person-based incidence was calculated until onset of the conscript's first MSD. Event-based incidence was calculated by dividing the total number of MSDs by the exposure time. Exposure time for event-based incidence was calculated until the end of follow-up. Time loss due to MSD was allowed for when calculating the exposure time for the event-based incidence. If a conscript was discharged from the military service, this was taken into account in exposure times. The incidences with 95% confidence intervals (CI) were expressed per 1000 person-days. Descriptive statistics were used to analyse the data. Cross-tabulations and chi-square test were used to analyse categorical variables. To examine differences in the occurrence rate of MSDs between the two arrivals of conscripts and between the service stages, the χ^2 ^statistics was used to test the hypothesis of no difference. Mann-Whitney U test was used to test if a difference existed between the arrivals in age variable. Since BMI was distributed normally, the difference of BMI between the arrivals was analysed by using the Independent t-test. A *P *value of < 0.05 was considered statistically significant.

## Results

### Occurrence of musculoskeletal disorders

During the 12-month study period (July 2006 – June 2007), altogether 437 outpatient clinic visits were registered in the garrison clinic due to MSDs. A total of 318 of 955 (33%) conscripts sustained one or several MSDs during the six-month service. Of these, 72% were treated once, 20% twice and 8% three or four times at the clinic. The event-based incidence for MSD was 3.3 (95% CI: 3.0–3.7) per 1000 person-days. Person-based incidence was 2.4 (95% CI: 2.2–2.6) per 1000 person-days.

Occurrence of MSDs was highest during the summer months with the peak in August (18 admissions per 100 conscripts) when the July arrivals were performing their intensive basic training period. In winter, the rates were generally lower with the lowest seen in March (3 admissions per 100 conscripts). No clear peak was found in January or February (8 and 7 admissions per 100 conscripts, respectively), when the second arrival served their first weeks. For the majority of conscripts military service has been divided into three stages of equal duration. During the first stage (basic training, service weeks 1–8), 15% of conscripts were treated at least once at the garrison clinic due to MSD. In the second (special training, service weeks 9–17) and third stages (team training, service weeks 18–26), the figures were approximately 14% and 13%, respectively. These rates were not statistically significantly lower compared to the rate of the basic training stage (χ^2^-test, *P *> 0.10 for both). However, the first arrival of conscripts (July 2006) had a higher occurrence rate for MSDs (40%) than the second arrival starting in January 2007 (29%) (χ^2^-test, *P *< 0.001).

### Type and anatomical location of musculoskeletal disorders

**Figure 2:**
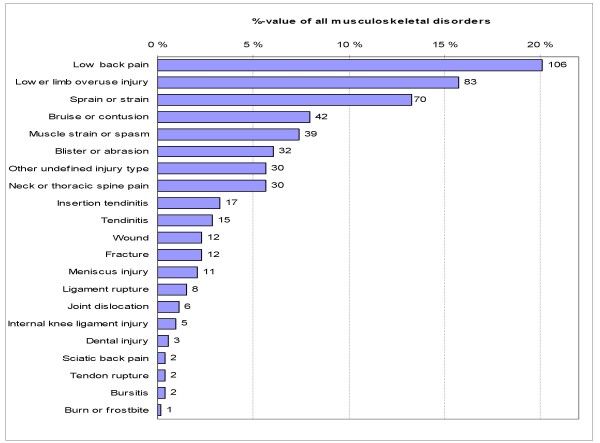
**Distribution of musculoskeletal disorders by injury type in 955 male conscripts during 6-month military service**. Count next to the bar is the absolute number.

**Figure 3:**
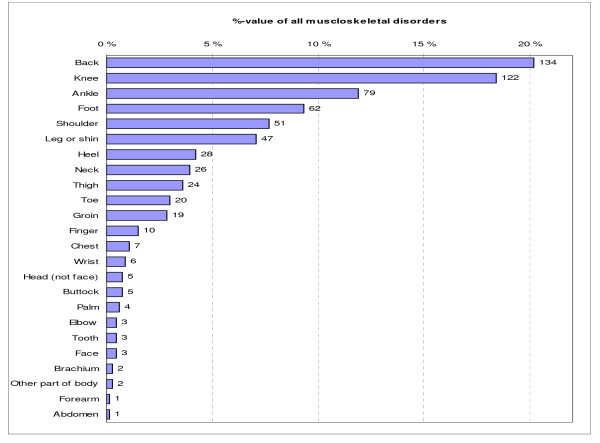
**Distribution of musculoskeletal disorders by anatomical location in 955 male conscripts during 6-month military service**. Count next to the bar is the absolute number.

The most common types of MSDs were LBP (20%), lower limb overuse injuries (16%) and sprains or strains (13%), which accounted for 49% of all disorders (Fig. 2). Most disorders were found on the lower limbs (61%). The upper limbs (including shoulders) were involved in 12% and the other parts of the body in 27% of the disorders. Anatomically, the most typical locations were the back (20%), the knee (18%), the ankle (12%), and the foot (9%), and they represented over half (60%) of all anatomical locations with MSDs (Fig. 3).

Overuse-related MSDs (66%) were nearly two times more prevalent than traumatic ones (34%). This distribution remained the same for both conscript batches. Foot and ankle disorders mostly originated from overuse (Table 3).

**Table 3:** Proportions of acute and overuse-related musculoskeletal disorders in 955 male conscripts during 6-month military service.

Body part	Acute		Overuse		Total Number
	n	%	n	%	
Back	43	33	89	67	132
Knee	42	35	78	65	120
Ankle	19	24	60	76	79
Foot	5	8	57	92	62
ALL BODY PARTS	146	34	281	66	427

The four most common body parts are shown in the table. In 10 cases the information considering the onset of the disorder remained unclear.

One third (34%) of the MSDs were recurrent disorders and 66% were new. Lower limb injuries or disorders in the ankle or foot were mostly new (84–87%), whereas disorders of the back and the knee were more frequently recurrent conditions (Table 4).

**Table 4:** Proportions of new and recurrent musculoskeletal disorders in 955 male conscripts during 6-month military service.

Body part	New		Recurrent		Total Number
	n	%	N	%	
Back	74	56	58	44	132
Knee	67	56	53	44	120
Ankle	66	84	13	16	79
Foot	54	87	8	13	62
ALL BODY PARTS	284	66	145	34	429

The four most common body parts are shown in the table. In 8 cases the information regarding recurrence of the disorder remained unclear.

### Associated activities and severity of musculoskeletal disorders

MSDs occurred mostly (91%) in the course of the military service, 9% during vacations and two cases (0.5%) occurred while travelling to holiday or back to the garrison.

Of the aetiological circumstances, combat training in combat gear was more common (40% of all scenes) than marching on foot or bicycle (28%) or other physical exercise (13%). In total, over 90% of the disorders emerging during military service were training-related (Fig. 1). Disorders during marching were mostly overuse type, whereas traumatic injuries were more common during combat training in combat gear or during other physical exercise (Table 5).

**Table 5:** Proportions of acute and overuse-related musculoskeletal disorders in 955 male conscripts during 6-month military service.

Associated activity	Acute		Overuse		Total Number
	n	%	N	%	
Combat training in combat gear	59	36	107	64	166
March on foot or by bicycle	8	7	110	93	118
During other physical exercise	29	54	25	46	54
ALL ASSOCIATED ACTIVITIES	146	34	282	66	428

Three most common associated activities are shown in the table. In 9 cases activities associated with musculoskeletal disorder remained unclear.

The majority (87%) of disorders were classified minor leading to a maximum of 7-day exemption from physical exercise, while moderate disorders accounted for 9% and severe disorders for 4% of all cases. Fractures, knee ligament ruptures, dislocations and muscle strains represented the most severe injuries and accounted for the highest number of service days lost. Seven of twelve fractures had traumatic origin (wrist (2 cases), brachium, finger, clavicle, foot and neck of the femur) and five were stress fractures (foot (4 cases), calcaneus). In addition, there were six dislocations (one patellar, one of the sternoclavicular joint and four anterior dislocations of the humerus).

Of the total of ninety discharges (9% of all conscripts), twenty-four (2.5%) conscripts were released temporarily (for at least 6 months) from military service due to musculoskeletal injuries consisting mostly of overuse injuries of the lower limb, LBP, tendinopathies and joint dislocations. All permanent releases (6 conscripts) were due to mental disorders (Table 2). Of these, three had a secondary diagnosis associated with permanent medical discharge. The associated diagnoses were M79.0 (unspecified rheumatism), J30 (vasomotor and allergic rhinitis) and F32.9 (unspecified depressive episode).

## Discussion

MSDs are an important cause of morbidity among Finnish conscripts. The occurrence rate of MSDs was 33% (or 333 per 1000 conscripts) during a six-month service period. Most MSDs involved the lower limb (61%), but LBP was also common. The high proportion of disorders affecting the low back and the lower limbs is noteworthy due to their commonly chronic nature causing time loss and premature releases from military service.

In the present study, the event-based incidence rate was 3.3 per 1000 person-days, which is slightly lower than in the two previous studies on conscripts [14,16]. Heir and Glomsaker (1996) monitored 6488 Army, Air Force and Navy conscripts during 6–10-wk period of military basic training in Norway and reported an incidence of approximately 4.2 per 1000 person-days for musculoskeletal injuries, including LBP. Rosendal et al. (2003) prospectively followed 330 Danish conscripts for 12 weeks in military basic training and reported an overall injury occurrence rate of 28% and a person-based incidence rate of approximately 3.5 per 1000 person-days. In the present study, complaints causing no time loss, like minor bruises, wounds and blisters not treated in the garrison clinic were not registered by medical staff, which may partly explain the difference in the occurrence rates between the studies. Also, the intensity of military training may be lower after the initial first weeks, which may be seen as lower injury rates during a longer follow-up time [20].

In this study, a peak of MSDs was seen during the basic training stage for conscripts arriving in July, but less clearly for those arriving in January. Since there were no significant differences between the batches considering the basic characteristics, it is suspected that this seasonal variation occurred due to environmental changes. Several explanations for the seasonal variation in the results may exist. Firstly, since the military training programmes for both arrivals were basically the same, winter may be a protective factor, as was also suggested in a previous Finnish conscript study [2]. A difference in strain may occur due to the winter environment when running and marching on foot are replaced by skiing which reduces the shock to the lower limbs. Also, snow, acting like a cushion, may reduce both traumatic and overuse-related MSDs. Knapik and colleagues [21] (2002) reported the same phenomenon indicating that injury incidence among US Army conscripts is higher in the summer than in the fall and suggested that environmental temperature may provide a partial explanation for the finding. In a large civilian study, a higher injury occurrence rate likewise appeared to be associated with higher environmental temperatures [22].

The high proportion of MSDs in the lower limb (61%) is consistent with the findings of several previous studies concerning military recruits [20,23-25] as well as conscripts in mandatory armies [2,14,16]. It seems that the military basic training exerts a load particularly on the lower limbs. Most conscripts are not used to marching long distances over rough terrains with a heavy load, which may be a factor behind overuse injuries [26]. According to a meta-analysis study, the best way to prevent lower limb fatigue fractures is to use shoes incorporating a proper shock absorbing cushion [27]. However, data concerning the use of custom-made or prefabricated insoles for reducing lower limb injuries in military recruits is conflicting [23,28-30]. Other methods proven to prevent physical activity-related injuries in randomised controlled trials include the use of external joint supports, neuromuscular training, controlled use of protective equipment, careful rehabilitation of injuries and gradual increase of physical exercise [23,29,31,32].

The high proportion of sprains, strains and lower limb overuse injuries is in accordance with previous studies [2,14,15,23,33,34]. Heir and Glomsaker (1996) reported similar results in Norwegian conscripts for LBP and knee overuse injuries. Hence, it seems that basic military training especially exposes conscripts to overuse injuries and LBP. In contrast, among the general population, only about 30% of physical activity-related injuries originate from overuse [35]. The observed high proportion of training-related disorders is in agreement with previous studies [30,36].

Considering that at the turn of the millennium a substantial rise (62%) was seen in the number of premature discharges due to MSDs [3], it was not surprising that MSDs and injuries emerged as an important cause for discharge in this study as well (27% of all premature discharges, Table 2). One explanation for the high occurrence of MSDs may be found in the changes implemented in the Finnish military service training programme in July 1998 which doubled the amount of physical exercise. On the other hand, the rise may be explained by conscripts being prematurely released from military service on minor grounds than before. In this study, 9% of all conscripts during the study year were prematurely discharged, which corresponds to the general level (8–10%) in the Finnish Defence Forces [3].

In the Finnish Defence Forces, the most common single reason behind medical discharges due to MSDs is LBP (21%), and the number of LBP-related discharges started to rise alarmingly in the late 1990s [3]. Chronic LBP is debilitating in military service and results in a notable increase in the use of health services [8]. However, severe low back disorders leading to hospitalisations are still rare in the early adulthood [37]. The present study indicated that a high proportion (44%) of back-related disorders were recurrent conditions and hence potential reasons for untimely discharge from military service. There is growing evidence that low back disorders occur where movement and motor control impairments appear as a result of abnormal tissue loading and pain. The consequences of these changes along with psychological and societal processes are potential factors behind the observed development [38-40]. Conscripts who suffer from chronic LBP before entering military service have a ten-fold higher risk to experience LBP during military service compared to the risk before the service [8]. This finding reflects the fact that basic military training is physically demanding for the back and requires an adequate level of physical fitness.

The mandatory military service in Finland differs from a recruit army system, such as in the United States, with respect to the number of conscripts, their quality and motivation, as well as the scope of the military programme. In a conscription army, the pace and content of military training have to be carefully adjusted to the fitness level of the conscripts. Combined with the short military service (180 days), this renders both the physical and military skill levels among conscripts lower than among their professional counterparts. Therefore, the results presented in this study cannot be directly extrapolated to a recruit army.

The present study had several strengths. First, the definition of MSD was clear and it was similarly understood by both the conscript himself and by the clinic physician or nurse, who treated and diagnosed the MSD and helped to fill the disorder questionnaire. Second, the participation rate was high (99%). Furthermore, the design of the study was a prospective follow-up of two successive batches of conscripts with the aim to provide information on the incidence of MSDs in an army environment during one whole year. The number of premature discharges (90 conscripts, 9%) from the military service during the study period may be considered a limitation of the study, as well as the descriptive nature of the study. In addition, since the threshold for seeking medical care may vary between individuals, some conscripts may have been more inclined to seek professional care than others.

The present study underlines the importance of MSDs as a cause of morbidity and premature discharge from military service in the Finnish Defence Forces. Given that the great majority (80%) of young men complete their military service in Finland, the high occurrence of MSDs in this population has an impact on public health. The current findings challenge the researchers and the military personnel to recognise and identify the risk factors in order to take preventive actions to decrease the number of MSDs among conscripts. Preventive measures during military service should be targeted at decreasing LBP and lower limb overuse injuries, because these represent the majority of MSDs and tend to have a chronic nature. The current best evidence for successful secondary prevention of LBP is provided by psychosocial and cognitive-behavioural interventions, as well as exercises enhancing motor control, flexibility and muscular strength and endurance of the trunk muscles [40-42]. However, as the efficiency of those programmes has not been well established, especially regarding early prevention of recurrence of LBP, more evidence is needed [42,43]. Knowledge of the risk factors and injury mechanism is an essential component for planning intervention programmes. The authors would recommend randomised controlled studies to provide more evidence from interventions before large scale prevention programmes are initiated in a military environment. In conclusion, preventive measures during military service should be targeted at decreasing LBP and lower limb overuse injuries, which are the largest burden among MSDs with tendency towards becoming chronic.

## Conclusion

In the present study two successive batches of physically active young conscripts were followed prospectively over a one year period. The observed high prevalence of MSDs in the lower back and lower limbs should be taken into account when planning prevention strategies. Fractures, knee ligament ruptures, dislocations and muscle strains accounted for the highest number of service days lost. Twenty-four (2.5%) out of 955 conscripts were prematurely discharged due to MSDs. Before initiating intervention programmes, risk factors and injury mechanisms leading to injuries and LBP need to be thoroughly assessed.

## Competing interests

The authors declare that they have no competing interests.

## Authors' contributions

HT participated in manuscript writing, data analysis, interpretation and data acquisition. JS was the primary investigator together with JP. She initiated and conceptually designed the study and took part in data processing and manuscript writing. HP participated in study concept and design as well as manuscript reviewing. VMM took part in data analysis and interpretation and gave statistical expertise. He also participated in the study as a significant manuscript reviewer. OO revised the manuscript critically and took part in data analysis and interpretation. He also participated in the study concept and design. PV took part in designing the study and data acquisition. He also revised the manuscript critically. JP was the primary investigator together with JS. He initiated and conceptually designed the study and participated in manuscript writing, data analysis and interpretation. All authors have made substantive intellectual contributions to the study. All authors reviewed the article and gave the final approval of the manuscript.

## Pre-publication history

The pre-publication history for this paper can be accessed here:


